# The Inhibitory Effect of Non-Steroidal Anti-Inflammatory Drugs (NSAIDs) on the Monophenolase and Diphenolase Activities of Mushroom Tyrosinase

**DOI:** 10.3390/ijms12063998

**Published:** 2011-06-14

**Authors:** Kazuomi Sato, Masaru Toriyama

**Affiliations:** 1 Department of Life Science, College of Agriculture, Tamagawa University, 6-1-1 Tamagawa-Gakuen, Tokyo, Japan; 2 Department of Applied Biological Science, Faculty of Agriculture, Shizuoka University, 836 Oya, Shizuoka, Japan; E-Mail: acmtori@ipc.shizuoka.ac.jp

**Keywords:** tyrosinase, monophenolase, diphenolase, NSAIDs (non-steroidal anti-inflammatory drugs), diflunisal, indomethacin

## Abstract

In the present work, we investigated the effect of non-steroidal anti-inflammatory drugs (NSAIDs) on the monophenolase and diphenolase activity of mushroom tyrosinase. The results showed that diflunisal and indomethacin inhibited both monophenolase and diphenolase activity. For monophenolase activity, the lag time was extended in the presence of diflunisal. In the presence of indomethacin, the lag time did not change. IC_50_ values of monophenolase activity were estimated to be 0.112 mM (diflunisal) and 1.78 mM (indomethacin). Kinetic studies of monophenolase activity revealed that both diflunisal and indomethacin were non-competitive inhibitors. For diphenolase activity, IC_50_ values were estimated to be 0.197 mM (diflunisal) and 0.509 mM (indomethacin). Diflunisal and indomethacin were also found to be non-competitive diphenolase inhibitors.

## 1. Introduction

Tyrosinase (monophenol monooxygenase; polyphenol oxidase; catechol oxidase; and oxygen oxidoreductase; E.C. 1.14.18.1) is a copper-containing enzyme, responsible for the formation of the melanin of skin, hair, and eye [1,2]. In addition, tyrosinase is found in animals, plants, fungi and microorganisms [1].

Tyrosinase catalyzes two different chemical reactions: the hydroxylation of monophenol and the oxidation of *O*-diphenol to the corresponding *O*-quinone [3–5]. Recently, the crystallographic structure of tyrosinase was reported, and this enzyme has a catalytic center formed by dinuclear copper [4,6,7]. Skin pigmentation results from melanin synthesis by melanocytes and is caused by exposure to UV radiation. Melanin plays an important role in the prevention of UV-induced skin injury and is a major determinant of skin color [8,9]. However, tyrosinase enhances melanogenesis, which leads to many hyperpigmentation disorders such as melasma, postinflammatory pigmentation, and solar lentigo, and these symptoms become prominent with aging. Moreover, tyrosinase is responsible for the undesired browning of fruits and vegetables during post-harvest storage [10,11]. Therefore, studies of inhibitory mechanisms of tyrosinase inhibitors are important in preventing enzymatic browning of vegetables and hyperpigmentation disorders. A huge number of tyrosinase inhibitors have been studied to date. For example, various aromatic carboxylic acids are known to be potent inhibitors of tyrosinase [12,13].

Non-steroidal anti-inflammatory drugs (NSAIDs) are known to act by directly suppressing the activity of cyclooxygenase (COX), the key enzyme catalyzing the biosynthesis of prostaglandins, which induce inflammation. Therefore, NSAIDs are generally used to treat pain, inflammation and fever. Moreover, some researchers reported that NSAIDs are able to prevent the development of cancer of the colon, breast, stomach and lung [14–17]. These biological effects of NSAIDs are the result of their ability to induce apoptosis and cell cycle arrest [18,19]. Acetylsalicylic acid is one of the most well studied NSAIDs, and has been shown to be effective as an antiplatelet drug in preventing heart attacks, stroke and cerebral thrombosis [20–22].

**Figure 1:**
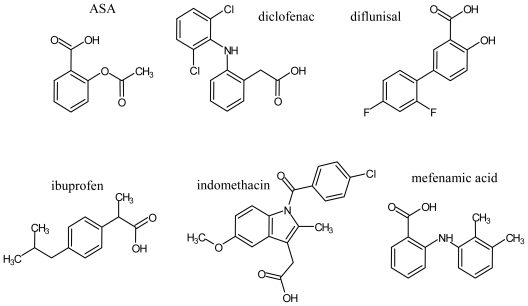
Chemical structure of NSAIDs used in this study.

Recently, we found that acetylsalicylic acid (ASA) down-regulates tyrosinase protein expression and inhibits melanogenesis on B16F1 melanoma cells [23], and we reported that mefenamic acid and diclofenac also inhibit melanogenesis [24]. However, no reports on the anti-melanogenic effect of NSAIDs have been published. Therefore, it is important to investigate the effects of other NSAIDs on melanogenesis. Consequently, we sought to investigate the inhibitory effect of NSAIDs (acetylsalicylic acid ASA, mefenamic acid, diclofenac, diflunisal, ibuprofen and indomethacin) (Figure 1) on the activity of mushroom tyrosinase. These NSAIDs are well-known non-selective COX inhibitors [25].

## 2. Results and Discussion

### 2.1. Effects of NSAIDs on the Monophenolase and Diphenolase Activity of Mushroom Tyrosinase

To clarify the effects of NSAIDs on mushroom tyrosinase activity, we first performed a tyrosinase assay to investigate monophenolase activity by using l-tyrosine as a substrate. As summarized in Table 1, ASA, ibuprofen and mefenamic acid did not inhibit monophenolase activity of tyrosinase. Diclofenac and mefenamic acid slightly inhibited tyrosinase activity (25% inhibition at the concentration of 4 mM diclofenac and 9% inhibition at 1 mM mefenamic acid, respectively). ASA did not show any inhibition. On the other hand, indomethacin showed inhibitory activity (IC_50_ = 1.78 mM), and furthermore diflunisal significantly inhibited tyrosinase activity (IC_50_ = 0.112 mM).

To investigate the effect of NSAIDs on diphenolase activity, we performed a tyrosinase assay by using l-DOPA as a substrate. ASA and ibuprofen did not inhibit diphenolase activity of mushroom tyrosinase (Table 1) (−6% inhibition at 4 mM ASA and 8% inhibition at 1 mM ibuprofen, respectively). Mefenamic acid slightly inhibited diphenolase activity at the concentration of 0.2 mM. Diclofenac slightly inhibited diphenolase activity (IC_50_ = 7.1 mM). Both diflunisal and indomethacin inhibited diphenolase activity. The IC_50_ values of these compounds were estimated at 0.197 mM and 0.509 mM, respectively. Diflunisal inhibited monophenolase activity more than diphenolase activity. For indomethacin, the IC_50_ value of the monophenolase activity was about three times larger than diphenolase activity.

In this study, we used non-selective COX inhibitors [25]. ASA, ibuprofen and mefenamic acid did not inhibit monophenolase or diphenolase activity. On the other hand, diclofenac, diflunisal and indomethacin inhibited both catalytic activities. These results imply that the inhibitory effect of COX activity of NSAIDs was not related to tyrosinase activity.

### 2.2. The Mechanism of Inhibition on Monophenolase Activity of Mushroom Tyrosinase by Diflunisal and Indomethacin

**Figure 2:**
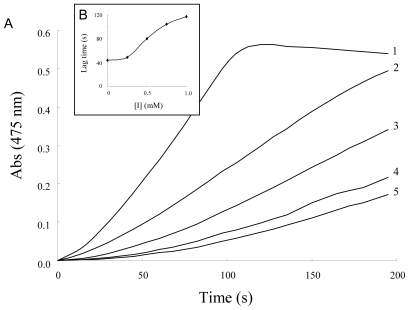
(**A**) Progress curves for the inhibition of monophenolase activity of tyrosinase by diflunisal. l-tyrosine was incubated with mushroom tyrosinase with or without diflunisal as described in the Experimental Section. The concentrations of diflunisal for curves 0–5 were 0 mM, 0.25 mM, 0.5 mM, 0.75 mM and 1.0 mM, respectively; (**B**) Relationship of the lag time with the concentration of diflunisal.

**Figure 3:**
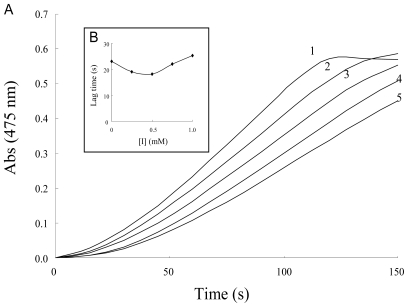
(**A**) Progress curves for the inhibition of monophenolase activity of tyrosinase by indomethacin. l-tyrosine was incubated with mushroom tyrosinase with or without indomethacin as described in the Experimental Section. The concentrations of indomethacin for curves 0–5 were 0 mM, 0.25 mM, 0.5 mM, 0.75 mM and 1.0 mM, respectively; (**B**) Relationship of the lag time with the concentration of indomethacin.

**Figure 4:**
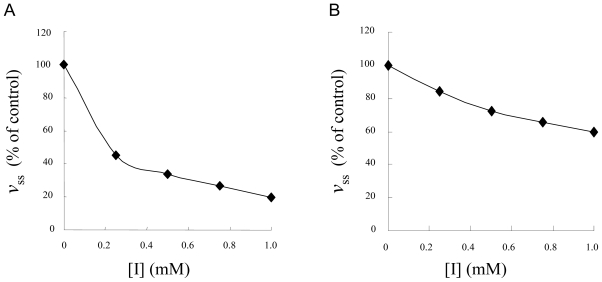
Relationship of the steady-state rate with the concentration of diflunisal (**A**) and indomethacin (**B**) l-tyrosine was incubated with mushroom tyrosinase with or without diflunisal or indomethacin as described in Experimental Section.

Several NSAIDs inhibit mushroom tyrosinase activity. We therefore investigated the inhibitory mechanism of diflunisal and indomethacin on mushroom tyrosinase. When the monophenolase activity of mushroom tyrosinase was assayed using l-tyrosine as a substrate, a lag time, characteristic of monophenolase activity, was observed simultaneously with the appearance of the first stable product, dopachrome, which was derived from oxidative hydroxylation of monophenolic substrates [25,26]. The kinetic course of the inhibition of monophenolase activity by different concentrations of diflunisal is shown in Figure 2. The results showed that diflunisal had an inhibitory effect on monophenolase activity and the system reached a steady state after the lag time. The lag time was extended in the presence of diflunisal in a dose-dependent manner. On the other hand, despite the fact that indomethacin inhibits the kinetic courses of monophenolase activity, the lag time did not change in the presence of inhibitor (Figure 3). In the presence of diflunisal, monophenolase activity of mushroom tyrosinase was significantly inhibited (approximately 45 ± 13% at 0.25 mM, 34 ± 7% at 0.5 mM, 27 ± 6% at 0.75 mM and 20 ± 8% at 1.0 mM) (Figure 4A). Indomethacin also inhibited monophenolase activity in a dose-dependent manner (approximately 84 ± 4% at 0.25 mM, 72 ± 5% at 0.5 mM, 66 ± 6% at 0.75 mM and 60 ± 4% at 1.0 mM) (Figure 4B).

**Figure 5:**
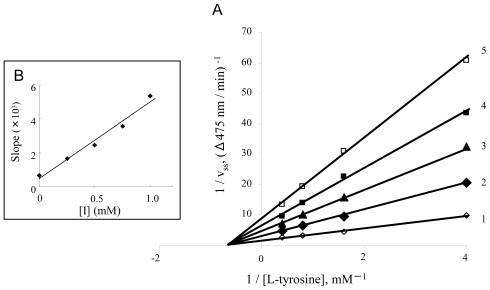
Determination of inhibitory type and inhibition constants of diflunisal on monophenolase activity of mushroom tyrosinase. (**A**) Lineweaver-Burk plots for inhibition of diflunisal on monophenolase activity. The concentrations of diflunisal for curves 1–5 were 0 mM, 0.25 mM, 0.5 mM, 0.75 mM and 1.0 mM, respectively; (**B**) The plots of slope *versus* the concentration of diflunisal.

**Figure 6:**
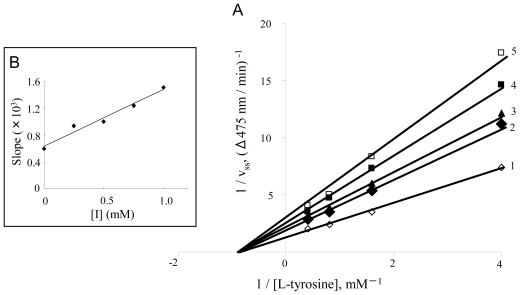
Determination of inhibitory type and inhibition constants of indomethacin on monophenolase activity of mushroom tyrosinase. (**A**) Lineweaver-Burk plots for inhibition of indomethacin on monophenolase activity. The concentrations of indomethacin for curves 1–5 were 0 mM, 0.25 mM, 0.5 mM, 0.75 mM and 1.0 mM, respectively; (**B**) The plots of slope *versus* the concentration of indomethacin.

We also investigated the inhibition type of diflunisal and indomethacin on the monophenolase activity of mushroom tyrosinase. Lineweaver-Burk plots for the inhibition of monophenolase activity by diflunisal were obtained (Figure 5A). The plots of 1/*V*ss *versus* 1/[S] gave a family of straight lines with different slopes which intersected one another on the X-axis, indicating that diflunisal is a non-competitive inhibitor. The equilibrium constants for inhibitor binding with the free enzyme and the enzyme-substrate complex, K_I_ and K_IS_, were obtained from the secondary plot (Figure 5B) as 0.11 mM and 0.11 mM. The kinetics of the enzyme in the presence of indomethacin are shown in Figure 6. The results showed that indomethacin was also a non-competitive inhibitor. The inhibitor constants (K_I_ and K_IS_) were estimated to be 0.75 mM and 0.75 mM, respectively.

### 2.3. The Mechanism of Inhibition on Diphenolase Activity of Mushroom Tyrosinase by Diflunisal and Indomethacin

**Figure 7:**
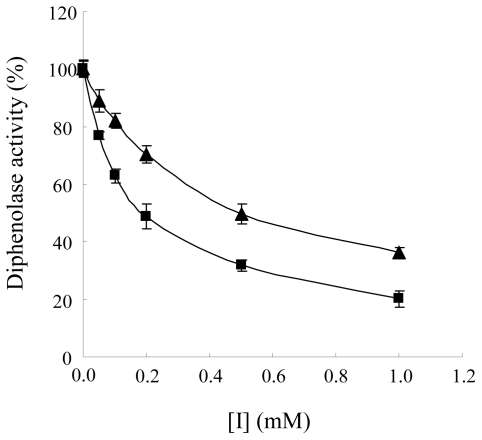
Effects of diflunisal (■) and indomethacin (▴) on the diphenolase activity of mushroom tyrosinase. l-DOPA was incubated with mushroom tyrosinase with or without diflunisal of indomethacin as described in the Experimental Section.

We also investigated the effect of diflunisal and indomethacin on diphenolase activity by using l-DOPA as a substrate. As shown in Figure 7, the result showed that both diflunisal and indomethacin inhibit the diphenolase activity in a dose-dependent manner. The IC_50_ values of diflunisal and indomethacin are shown in Table 1. In the presence of diflunisal, diphenolase activity of mushroom tyrosinase was clearly inhibited (approximately 77 ± 1% at 0.05 mM, 63 ± 2% at 0.1 mM, 49 ± 4% at 0.2 mM, 32 ± 2% at 0.5 mM and 20 ± 2% at 1.0 mM) (Figure 7). Indomethacin also inhibited diphenolase activity in a dose-dependent manner (approximately 89 ± 4% at 0.05 mM, 82 ± 3% at 0.1 mM, 70 ± 3% at 0.2 mM, 50 ± 3% at 0.5 mM and 32 ± 2% at 1.0 mM).

**Figure 8:**
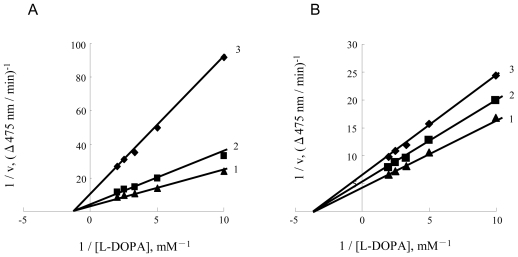
Determination of inhibitory type of diflunisal and indomethacin on diphenolase activity of mushroom tyrosinase. (**A**) Lineweaver-Burk plots for inhibition of diflunisal on diphenolase activity. The concentrations of diflunisal for curves 1–3 were 0.1, 0.2 and 1.0 mM, respectively; (**B**) Lineweaver-Burk plots for inhibition of indomethacin on diphenolase activity. The concentration of indomethacin for curves 1–3 were 0.1, 0.2 and 1.0 mM, respectively. The results showed that indomethacin also was a non-competitive inhibitor.

We studied the inhibitory mechanism of diflunisal and indomethacin on diphenolase activity of mushroom tyrosinase. Lineweaver-Burk plots for inhibition of diflunisal are shown in Figure 8A. The results showed that diflunisal was a non-competitive inhibitor since the plots of 1/v *versus* 1/[S] gave a family of straight lines with different slopes, which intersected one another in the X-axis. The inhibitor constants (K_I_ and K_IS_) were estimated to be 0.19 mM and 0.19 mM, respectively. The kinetics of the enzyme in the presence of indomethacin is shown in Figure 8B. The results showed that indomethacin also was a non-competitive diphenolase inhibitor. The inhibitor constants (K_I_ and K_IS_) were estimated to be 2.42 mM and 2.42 mM, respectively.

## 3. Experimental Section

Tyrosinase from mushroom was purchased from Sigma. Dimethylsulfoxide (DMSO), l-tyrosine, l-3,4-dihydroxyphenylalanine (l-DOPA), acetylsalicylic acid (ASA), mefenamic acid, diclofenac, diflunisal and kojic acid were purchased from Sigma. Ibuprofen and indomethacin were purchased from Wako Pure Chemical. All NSAIDs and kojic acid used in the monophenolase and diphenolase activity assay were dissolved in DMSO.

The monophenolase activity assay was performed with modification as reported by Chen *et al*. [27]. In this assay, l-tyrosine was used as substrate in the monophenolase activity assay. First, 300 μL of 2.5 mM l-tyrosine, 25 μL of DMSO with or without a sample chemical, and 300 μL of 0.1 M phosphate buffer (pH 6.8) were mixed. The mixture was preincubated at 37 °C for 10 min before 125 μL of 1000 units/mL mushroom tyrosinase in aqueous solution added, and the reaction was performed at 37 °C. Enzyme activity was monitored at 475 nm by a UV spectrophotometer (Shimadzu, UV-1700). The lag time was determined by extrapolation of the linear portion of the dopachrome accumulation curve to the abscissa axis.

The diphenolase activity assay was performed as previously described [28]. First, 300 μL of 2.5 mM 3,4-dihydroxyphenylalanine (l-DOPA) solution, 50 μL of DMSO with or without a sample chemical, and 1.1 mL of 0.1 M phosphate buffer (pH 6.8) were mixed. The mixture was preincubated at 25 °C for 10 min before 50 μL of 1000 units/mL mushroom tyrosinase in aqueous solution was added, and the reaction was monitored at 475 nm. A control reaction was conducted in DMSO alone. The percentage of activity of tyrosinase was calculated as follows: 100 − [(A − B)/A × 100], where A represents the difference in the absorbance of the control sample between an incubation time of 0.5 and 1.0 min, and B represents the difference in the absorbance of the test sample over the same incubation period.

The inhibition type of NSAIDs on the monophenolase and diphenolase activity was determined by the Lineweaver-Burk plots and the inhibition constants were determined from secondary plots of the apparent Km/Vmax *versus* the concentration of the inhibitor.

## 4. Conclusions

In this study, we investigated the inhibitory effect of NSAIDs on mushroom tyrosinase. The results showed that diflunisal and indomethacin significantly inhibit monophenolase and diphenolase activity. As summarized in Table 2, the IC_50_ value of diflunisal on monophenolase activity was smaller than diphenolase activity. On the other hand, the IC_50_ value of indomethacin on monophenolase activity was about three times as large as diphenolase activity. The kinetic studies showed that both compounds were non-competitive inhibitors. We also estimated lag time on monophenolase assay. From the results, diflunisal lengthens the lag period. On the other hand, indomethacin did not lengthen the lag period. Li *et al.* described that if an inhibitor is combined with free enzyme, it lengthens the lag period. If an inhibitor is combined with ES (enzyme-substrate complex), it only decreases the steady-state rate but does not lengthen the lag period [29].

Our results showed diflunisal is the most effective tyrosinase inhibitor out of six NSAIDs in the present study. Interestingly, diflunisal is a chemical analog of ASA. However, ASA did not inhibit either monophenolase or diphenolase activity. Further studies of other NSAIDs on tyrosinase activity and the depigmenting effect of NSAIDs are very important. These compounds may be useful inhibitors of tyrosinase and they might lead to an effective treatment of hyperpigmentation disorders.

## Figures and Tables

**Table 1:** IC_50_ values of NSAIDs on monophenolase activity and diphenolase activity.

IC_50_ (mM)		
Compound	Monophenolase Activity	Diphenolase Activity
Acetylsalicylic acid	>4	>4
Diclofenac	>4	7.1
Diflunisal	0.112	0.197
Ibuprofen	>4	>1
Indomethacin	1.78	0.509
Mefenamic acid	>1	>0.2

**Table 2:** Inhibition constants of diflunisal and indomethacin with mushroom tyrosinase.

Constant		Diflunisal	Indomethacin
IC_50_	Monophenolase	0.112 mM	1.780 mM
	Diphenolase	0.197 mM	0.509 mM
Inhibition type		Non-competitive	Non-competitive
K_I_	Monophenolase	0.11 mM	0.75 mM
	Diphenolase	0.19 mM	2.42 mM
